# Exploring the Interaction of Biotinylated FcGamma RI and IgG1 Monoclonal Antibodies on Streptavidin-Coated Plasmonic Sensor Chips for Label-Free VEGF Detection

**DOI:** 10.3390/bios14120634

**Published:** 2024-12-20

**Authors:** Soodeh Salimi Khaligh, Fahd Khalid-Salako, Hasan Kurt, Meral Yüce

**Affiliations:** 1 SUNUM Nanotechnology Research and Application Centre, Sabanci University, Istanbul 34956, Türkiye; soodeh.salimi@sabanciuniv.edu (S.S.K.); fahd.khalidsalako@sabanciuniv.edu (F.K.-S.); 2 Department of Bioengineering, Royal School of Mines, Imperial College London, London SW7 2AZ, UK

**Keywords:** VEGF biosensor, FcγRI, monoclonal antibodies, surface plasmon resonance (SPR), biosensing technology

## Abstract

Vascular endothelial growth factor (VEGF) is a critical angiogenesis biomarker associated with various pathological conditions, including cancer. This study leverages pre-biotinylated FcγRI interactions with IgG1-type monoclonal antibodies to develop a sensitive VEGF detection method. Utilizing surface plasmon resonance (SPR) technology, we characterized the binding dynamics of immobilized biotinylated FcγRI to an IgG1-type antibody, Bevacizumab (AVT), through kinetic studies and investigated suitable conditions for sensor surface regeneration. Subsequently, we characterized the binding of FcγRI-captured AVT to VEGF, calculating kinetic constants and binding affinity. A calibration curve was established to analyze the VEGF quantification capacity and accuracy of the biosensor, computing the limits of blank, detection, and quantification at a 95% confidence interval. Additionally, the specificity of the biosensor for VEGF over other protein analytes was assessed. This innovative biomimetic approach enabled FcγRI-mediated site-specific AVT capture, establishing a stable and reusable platform for detecting and accurately quantifying VEGF. The results indicate the effectiveness of the plasmonic sensor platform for VEGF detection, making it suitable for research applications and, potentially, clinical diagnostics. Utilizing FcγRI-IgG1 antibody binding, this study highlights the industrial and clinical value of advanced biosensing technologies, offering insights to enhance therapeutic monitoring and improve outcomes in anti-VEGF therapies.

## 1. Introduction

Vascular endothelial growth factor (VEGF) promotes the proliferation and survival of endothelial cells while enhancing vascular permeability [1], thereby meeting the metabolic needs of the growing tumor. Due to the critical role of angiogenesis in tumor biology, drug development over the past decades has focused on targeting this process, with VEGF identified as a primary therapeutic target for inhibiting angiogenesis and normalizing tumor vasculature [2,3]. The crucial role of VEGF in tumor initiation, growth, and metastases also makes it a key serum biomarker of clinical significance in various cancers [4]. VEGF has demonstrated substantial clinical potential in early diagnostic predictions of treatment response, disease relapse, and prognosis. In recent studies, serum VEGF levels outperformed conventional cancer biomarkers like carcinoembryonic antigen (CEA), cancer antigen–125 (CA–125), and cytokeratin 19 fragments (Cyfra 21-1) in the early diagnosis and severity assessment of non-small cell lung cancers and ovarian cancer and also as a predictive biomarker for treatment response and prognosis in breast cancer [5,6,7]. These findings demonstrate the importance of early VEGF detection and monitoring in clinical decision making. Additionally, VEGF has been indicated as an important biomarker in other chronic diseases, such as rheumatoid arthritis [8], retinopathy [9], and Parkinson’s Disease [10], among others.

Several techniques have been adopted and reported in the literature for the detection of VEGF, including enzyme-linked immunosorbent assays (ELISA) [11], spectrofluorometry [12], and radioimmunoassay [13]. These conventional techniques are often cumbersome, requiring multiple steps in sample preparation, and are time-consuming. In contrast, surface plasmon resonance (SPR) biosensors provide a sensitive alternative, achieving sensitivity at the picomolar level [14].

The real-time, label-free detection possible in SPR assays provides a deeper understanding of biomolecular interactions based on longitudinal binding data. SPR-based sensors can specifically and accurately detect targeted biomolecules in biological fluids, including blood, urine, saliva, and plasma, even at low concentrations [14,15,16,17]. Therefore, SPR-based biosensors have been effectively employed for detecting antibodies, proteins, therapeutics, viruses, and nucleic acids [18,19] and for characterizing interactions between protein molecules [20].

The value of plasmonic sensors in achieving higher sensitivity and practicality—especially in cancer biomarker detection—is highlighted by recent promising findings emanating from VEGF biosensor setups such as Raman Spectroscopes and Luminescence Complexes with integrated plasmonic signal enhancement [21,22]. Similarly, there continues to be an uptrend in SPR-based biosensing setups. Recent advances improve the traditional Kretschmann configuration with detection and signal transduction enhancements, big data management, and analysis functionalization, among others [23]. Accordingly, there have been recent attempts to adapt SPR as a biosensor technique for cancer biomarker detection [24,25], immunoassays [26], and SARS-CoV-2 detection [27,28], among several other diagnostic applications [29]. Interestingly, the biosensing applications of SPR transcend disease diagnosis and monitoring, with researchers from IFM, Linköping University, Sweden, demonstrating a smartphone-based imaging SPR technique for allergen detection in food products [30].

In physiological systems, antibodies mediate immunologic activities through highly specific interactions with target antigens via their antigen-binding region (Fab) and recruitment of cellular immunity by binding epitopes on their crystallizable fragment (Fc) region, which interact with Fc receptors on the effector cells. Specifically, of all Fc Gamma Receptors (FcγRs), FcγRI is unique in its high IgG affinity, making it the only FcγR subclass capable of binding IgG in their monomeric state and without them being in complex with antigens [31,32]. Attempts to explain the relatively high binding affinity of FcγRI have suggested its distinguishing third domain and structurally peculiar features in the FG region, which interacts with antibodies Fc regions [33,34]. This unique property of FcγRI has significant implications for SPR assay development, as demonstrated by Dorion-Thibaudeau et al., who demonstrated the unsuitability of low-affinity FcγR subclasses in SPR biosensing setups due to their susceptibility to confounding factors and a potential low signal-to-noise ratio [35]. Our group already demonstrated the potential of FcγRI for site-oriented IgG1-type monoclonal antibody (mAb) capture in SPR-based immunologic assays, providing an opportunity to repurpose mAbs for biosensing applications through FcγRI capture [36].

The specificity of mAbs makes them powerful tools in diagnostics, therapeutics, and research [37,38]. They represent one of the most rapidly developing biotherapeutic classes in the global pharmaceutical industry. Widely used therapeutic mAbs include Bevacizumab (Avastin^TM^), a recombinant humanized mAb that mediates cytotoxicity by inhibiting VEGF-mediated angiogenesis. Bevacizumab specifically disrupts the interaction between human VEGF and endothelial cell surface receptors, thereby inhibiting VEGF’s biological activity and restricting angiogenesis [39]. In vivo studies have shown that bevacizumab inhibits blood vessel growth, induces the regression of newly formed vessels, and normalizes vascular structure, ultimately improving the delivery of cytotoxic chemotherapy [2].

**Figure 1:**
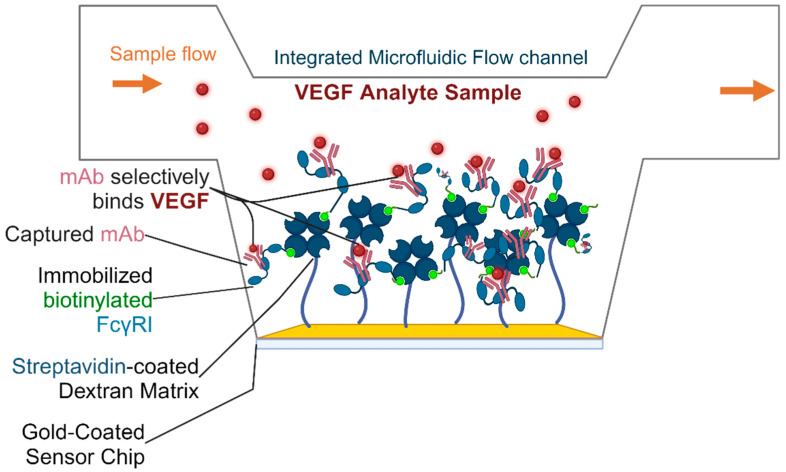
A schematic of the VEGF SPR biosensor setup. Biotinylated FcγRI is immobilized on the streptavidin-coated sensor surface. The mAb is captured by the immobilized FcγRI through high-affinity interactions with the mAb Fc regions, orienting the mAb Fab regions for the optimal detection and quantification of VEGF. Created in BioRender. Khalid-Salako, F. (2024) https://BioRender.com/g42b753.

In this study, we optimize an SPR-based biosensing setup for the efficient detection of VEGF, drawing on interactions of FcγRI-captured Bevacizumab (AVT) with VEGF. Accordingly, our approach is to recapitulate physiologic antibody conformations, using biotinylated FcγRI immobilized on a streptavidin-coated biosensor chip for the site-specific capture of AVT, the target-specificity of which theoretically provides clinical potential in the detection and quantification of VEGF analytes. The biosensor setup is illustrated in Figure 1. This method aggregates characteristic surface binding affinity and kinetics data on protein interactions to proffer valuable insights, potentially for clinical decision making.

## 2. Materials and Methods

### 2.1. Materials

The Biacore T200 instrument (Cytiva, Marlborough, MA, USA) was used for real-time biomolecular interaction analysis. Sensor chip SA with pre-coated streptavidin on carboxymethylated dextran matrix, sourced from Cytiva (Marlborough, MA, USA), was utilized for the experimental procedure. The ligand, FcγRI (Biotinylated Human CD64 Protein, His, Avitag^TM^), was acquired from ACRO Biosystems AG (Basel, Switzerland).

The monoclonal antibody Avastin (Roche) was prepared with 1x HBS-EP running buffer. VEGF (VEGF 165, human recombinant, >95% purity) was purchased from SIGMA (Darmstadt, Germany).

The running buffer (1x HBS-EP) was prepared using 10 mM HEPES, 150 mM NaCl, 3 mM EDTA, and 0.005% *v*/*v* Surfactant P20, with a pH of 7.4, using distilled water. The distilled water was autoclaved and filtered through a 0.22 μm membrane filter (Nitrocellulose [NC], GVS) before use. All experiments were conducted at 22 °C.

### 2.2. FcγRI Immobilization Procedure

The Sensor chip SA with streptavidin pre-coated on a carboxymethylated dextran matrix surface functionalization was inserted and docked on the Biacore T200 instrument (Cytiva, Marlborough, MA, USA), which was then primed following the manufacturer’s instructions. Subsequently, the sensor surface was conditioned with three consecutive 60 s injections of 1M NaCl and 50 mM NaOH solution at a 10 μL·min^−1^ flow rate, preparing the surface for subsequent protein binding by removing potential contaminants and stabilizing the baseline. Next, the biotinylated FcγRI was injected into the sensor chip’s flow channel in pulses following the preset immobilization wizard, with the immobilization target set to 200 Response Units (RU). This controlled immobilization ensures consistent binding levels across experimental repeats. To ensure the removal of any non-specifically bound materials and to stabilize the immobilized surface, the flow system was then washed with a 50% solution of isopropanol in 50 mM NaOH/1M NaCl [40].

### 2.3. Antibody Binding Assays

The binding assays were assessed using an FcγRI-immobilized surface. Five sequential concentrations of AVT (1.875, 3.75, 7.5, 15, and 30 nM) were injected for 60 s at a 50 μL·min^−1^ flow rate over both blank (reference) and active (FcγRI-immobilized) flow channels, allowing for the precise subtraction of non-specific binding from the resulting data.

Kinetic parameters were calculated using the Biacore Evaluation software (3.0 Biacore T200, Shrewsbury, MA, USA), applying the heterogeneous ligand binding kinetics model. K_D_ values from affinity analyses were determined using the software’s steady-state affinity algorithm. The results were acquired using the double referencing method, in which the presented data were adjusted by subtracting both the zero-concentration sample and the blank reference surface.

### 2.4. Sensor Surface Regeneration

The SPR biosensor operates on the principle of detecting changes in the refractive index of the superstrate. Molecular binding on the sensor surface is crucial for its functionality in sensing applications. Sensor surface regeneration is essential in SPR to ensure that any bound analytes or molecules from previous interaction cycles are cleared from the sensor surface. This regeneration step prepares the surface for new binding events, allowing multiple binding and dissociation cycles on the same sensor without interference from residual molecules. Accordingly, regeneration conditions were evaluated in two phases: scouting and verification to assess the reusability of the sensor surface and establish optimal regeneration conditions.

In the regeneration scouting phase, fourteen regeneration solutions were tested, including 50 mM phosphate buffer and 50 mM citrate buffer (both at pH 3), 10 mM acetate buffer (at pH 3, 4.0, 4.5, 5.0, and 5.5), 10 mM glycine hydrochloride (at pH 3, 2, and 1.5), ethylene glycol, 5 M NaCl, 4 M MgCl₂, and 0.5% sodium dodecyl sulfate (SDS). Each regeneration solution was applied to the sensor surface for five consecutive cycles of 30 nM AVT injection over the FcγRI-immobilized sensor surface. Trends in baseline and binding responses at equilibrium over the five cycles were observed as a basis for assessing the conditions’ surface regeneration efficacy and ligand activity preservation. The solutions demonstrating the most effective regeneration with minimal damage to ligand binding activity were selected and evaluated in separate cycles during regeneration verification.

For regeneration verification, 15 cycles of 30 nM AVT injections were performed, each lasting 60 s at a flow rate of 30 μL·min^−1^, over the blank (reference) and active (FcγRI-coated) flow cells. The surface was regenerated after each cycle, and the regeneration conditions were assessed. Relative responses obtained in each cycle were normalized against responses in the blank and active flow channels, and trends in normalized relative responses were monitored over the 15 cycles for each regeneration condition. The solution demonstrating the most effective sensor surface regeneration with minimal ligand activity damage, characterized by the lowest change in normalized relative responses from the first to the fifteenth cycle, was employed to regenerate the sensor surface for the rest of the study.

Further studies were subsequently conducted to ascertain sensor surface stability and characterize changes to sensor performance in successive cycles. Having immobilized biotinylated FcγRI at a 200RU immobilization target on the streptavidin-coated sensor surface, a 30 nM AVT solution was injected onto the sensor surface at a 30 µL·min^−1^ flow rate for 60 s to capture the mAb over the surface for 25 successive cycles. During cycles 5, 7, 9, 11, 13, 15, 17, 19, 21, and 23, a 30 nM VEGF solution was injected onto the sensor surface, following AVT capture, while blank buffer injections were carried out for the other cycles. Sensor surface stability was assessed as a measure of baseline, AVT capture, and VEGF binding over the successive cycles within a single run in which the sensor surface was regenerated with 10 mM glycine (pH 3), as informed by the regeneration study findings.

### 2.5. Antibody/Antigen Interaction Assays

#### 2.5.1. Antigen-Binding Assays

To evaluate the binding interactions, AVT was injected over the active and blank flow cells at a concentration of 30 nM and captured on the FcγRI-immobilized chip surface for 60 s. Subsequently, three sequential concentrations of VEGF (3.33, 10, and 30 nM) were injected with a 60-s association time for each concentration, followed by a 300-s dissociation time under a flow rate of 50 μL·min^−1^ at 22 °C. The sensor surface was regenerated with 10 mM glycine-HCl (pH 3) for 60 s.

Kinetic parameters were calculated using the Biacore Evaluation software (3.0 Biacore T200, Shrewsbury, MA, USA), applying the two-state binding model. The dissociation constant (KD) values were determined via affinity analysis using the steady-state affinity algorithm in the Biacore Evaluation software, providing a robust assessment of binding strength across the tested VEGF concentrations.

#### 2.5.2. Antigen Concentration Analyses

Following AVT capture on the FcγRI-immobilized chip surface, sequential injections of VEGF protein solutions at five distinct concentrations (1.1, 3.3, 10, 30, and 90 nM) were introduced over both the active and reference flow cells to evaluate and quantify the binding interaction across a range of VEGF levels. This analysis is essential for establishing a calibration curve for the precise quantification of VEGF in potentially unknown samples. Each injection cycle incorporated a 60-s association time, allowing VEGF to bind to the immobilized mAb, and a 300-s dissociation time to observe the decay in the binding signal. The experiment was conducted at a controlled flow rate of 50 μL·min^−1^ and a temperature of 22 °C. To restore the sensor surface between cycles, a regeneration step was performed using 10 mM glycine-HCl (pH 3) for 60 s, effectively preparing the surface for subsequent injections.

The calibration curve was generated using a four-parameter logistic fitting function on the Origin 2024b software (OriginLab Corporation, Northampton, MA, USA). The limit of detection (LOD) was determined as the concentration on the curve, corresponding to 3.3 times the standard deviation (*SD*) of replicate measurements from blank samples [41]. The accuracy and precision of the assay were calculated based on the standard deviation (*SD*), coefficient of variation (*CV%*), and accuracy/recovery values, as shown in Equations (1) and (2). The results were acquired using the double referencing method, in which the presented data were adjusted by subtracting both the zero-concentration sample and the blank surface.

(1) $$CV\%=\frac{SD}{mean}\times 100$$

(2) $$\frac{Accuracy}{recovery}\%=\frac{Calculated\;mean\;of\;AVT}{Theoretical\;mean\;of\;AVT}\times 100$$

### 2.6. Specificity Assays

To assess the specificity of surface FcγRI-captured AVT for its target antigen, VEGF, a specificity analysis was conducted by injecting three different antigens, including TNF-α (Tumor Necrosis Factor-alpha), HER2 (Human Epidermal Growth Factor Receptor 2), and VEGF, each at a concentration of 30 nM, alongside BSA (Bovine Serum Albumin) at 0.1 mg·mL^−1^ as a non-specific protein control. These proteins were selected to evaluate potential non-specific binding and confirm that a binding response occurs specifically between AVT and VEGF, rather than with irrelevant proteins. If a binding response is observed exclusively with VEGF and not with the control proteins, it confirms the specificity of the VEGF-AVT binding. This analysis is essential for validating that AVT selectively binds to VEGF over other proteins, a crucial characteristic for ensuring its therapeutic effectiveness and minimizing off-target interactions.

The prepared samples were injected sequentially over the active and blank surfaces of the FcγRI-immobilized chip to monitor binding interactions. Each injection involved a 60-s association time, allowing the antigens to interact with the immobilized Avastin, followed by a 300-s dissociation time at a flow rate of 50 μL·min^−1^ at 22 °C. The sensor surface was regenerated with 10 mM glycine-HCl (pH 3) for 60 s, and the results were processed using the double referencing method, in which the presented data were adjusted by subtracting both the zero-concentration sample and the blank surface.

## 3. Results

### 3.1. FcγRI Immobilization

Sensor chip SA utilizes the high-affinity (K_D_ ≈ 10^−15^ M) binding of streptavidin to biotin to effect the highly stable binding of biotinylated ligands to covalently linked streptavidin on the chip surface dextran matrix [42]. For versatility, it is necessary for the proposed biosensor setup to withstand surface regeneration and the attachment of new IgG–1-type mAbs for adjacent biosensing applications with minimal impediment to surface ligand binding capacity. The high-affinity streptavidin–biotin-binding ensures surface stability and maintains the mAb-binding activity of the FcγRI ligand demonstrable in steady sensorgram baselines produced over the course of successive runs (Figure S1). The stability and reproducibility of biotin capture, therefore, were preferred over other immobilization techniques, especially in a biosensor setup [43,44].

**Figure 2:**
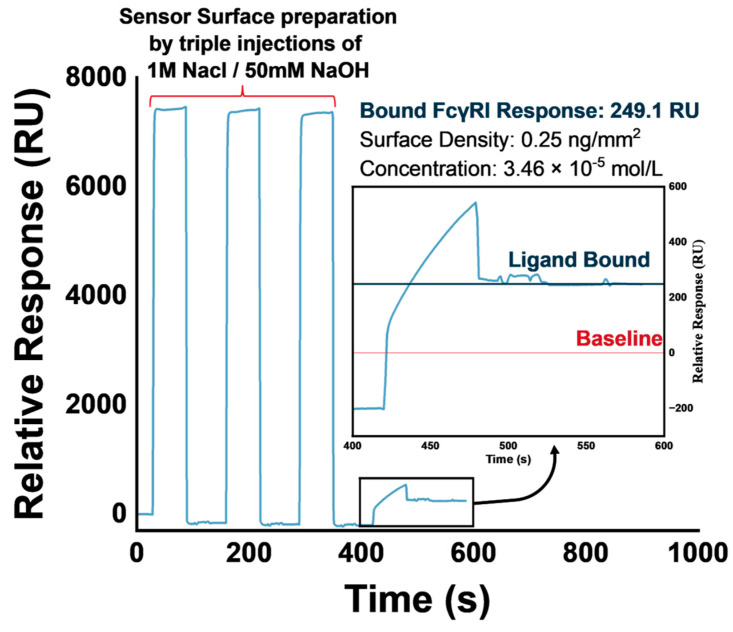
Sensorgram of biotinylated−FcγRI immobilization on streptavidin-coated SPR sensor chip.

Multiple immobilization runs were conducted throughout the study, using the SA-Biotin capture method to immobilize biotinylated FcγRI on the streptavidin-coated SPR sensor chip surface. A typical immobilization run sensorgram obtained is presented in Figure 2.

The immobilization levels of biotinylated-FcγRn (100 RU) and his-tagged FcγRI (200 RU) reported in the literature are similar to the immobilization levels achieved in our study [20,36]. We set a targeted immobilization level of 200 RU, achieving between 30.9 and 249.1 RU (Figure 2).

### 3.2. Regeneration

Adopting the streptavidin–biotin capture technique for immobilizing FcγRI allowed us to recapitulate the stable, high-affinity binding of FcγRI to IgG1-type antibodies. However, using a biotinylated FcγRI ligand on a streptavidin surface for IgG binding presents significant sensor surface regeneration challenges that must be addressed before our setup can be applied for biosensing applications. As encountered in an earlier study from our group [36], biotinylated FcγRI immobilized chip surfaces progressively lost IgG binding capacity. Additionally, the baseline increased with subsequent assays, suggesting incomplete regeneration and accumulation of IgG. Additionally, previous studies suggest that structural conformations that confer the high binding affinity of FcγRI for IgG make it impossible to regenerate the ligand from the complex without disrupting its structure and activity [45,46]. Therefore, it was important to sufficiently regenerate the biotinylated FcγRI to optimize our setup for biosensing applications without significantly disrupting its binding activities. This would enable the setup to be reliably applied to multiple mAb–antigen pairs. To achieve this, we studied various regeneration conditions in two phases: regeneration scouting and regeneration verification.

**Figure 3:**
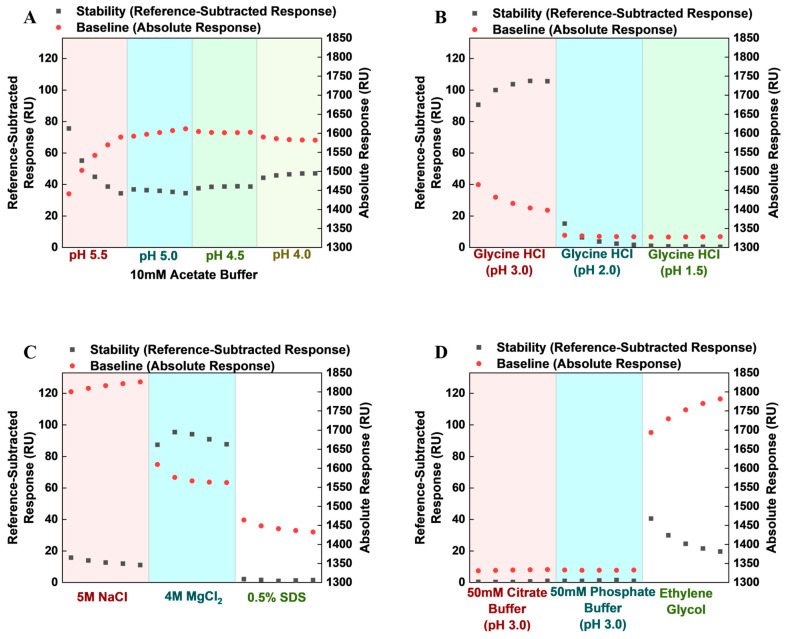
Baseline and stability trends over five repeated cycles as observed in (**A**) 10 mM acetate buffer pH 5.5, 5, 4.5, and 4.0; (**B**) glycine hydrochloride pH 3.0, 2.0, and 1.5; (**C**) 5 M NaCl, 4 M MgCl_2_, and 0.5% SDS; (**D**) 50 mM citrate buffer (pH 3.0), 50 mM phosphate buffer (pH 3.0), and ethylene glycol.

In regeneration scouting, we studied 14 solutions, including 50 mM phosphate buffer (pH 3); 50 mM citrate buffer (pH 3); 10 mM acetate buffer (pH 3, 4.0, 4.5, 5.0, and 5.5); 10 mM glycine hydrochloride (pH 3, 2, and 1.5); ethylene glycol; 5 M NaCl; 4 M MgCl_2_; and 0.5% sodium dodecyl sulfate (SDS). We assessed response trends (AVT binding capacity) and baseline (regeneration effectiveness) across five repeated analyte injection and regeneration cycles within each condition. Trends in response and baseline changes observed for the regeneration conditions studied are illustrated in Figure 3.

Visually examining the trends in stability/equilibrium response (R_eq_) and baseline, we determined that 10 mM acetate buffer at pH 5.5 was not optimally effective at regenerating the sensor surface, demonstrating a marked progressive increase in baseline response and a corresponding decline in R_eq_. This appears to be the case—although to a lesser extent, for pH 5.0, ethylene glycol, and NaCl. Over the five cycles, during which 4 M MgCl_2_ was used as the regeneration solution, both baseline and R_eq_ declined (the latter having initially had a sharp peak). With SDS, we found that the baseline decreased progressively while R_eq_ remained fairly stable. In the case of glycine HCL, pH 2 appeared to disrupt ligand activity, causing a decline in R_eq_, even at a relatively stable baseline response. Based on these findings, we proceeded to conduct regeneration verification exercises for glycine HCL (pH 3), 10 mM acetate buffer (pH 4.5), 50 mM citrate buffer (pH 3.0), and 50 mM phosphate buffer (pH 3), each over 15 cycles. Normalized relative response at equilibrium (_N_R_eq_) was calculated by computing all blank reference and sample relative responses (R^i^_eq_ = R_eq_ − R_baseline_) in reference and active flow channels. Subsequently, minimum and maximum R^i^_eq_ values (R_MIN_ and R_MAX_) were determined for each regeneration condition, and _N_R_eq_ was calculated using Equation (3) below.

(3) ReqN=Rieq−RMINRMAX−RMIN×100

Similarly, double-referenced R^i^_eq_ values were calculated for each cycle by subtracting R^i^_eq_ in the active flow channel from R^i^_eq_ in the blank reference channel, and _N_R_eq_ was calculated for the double-referenced R^i^_eq_ values. The regeneration conditions were compared using the difference in the normalized double-referenced R^i^_eq_ obtained from the first to the fifteenth cycle under each regeneration condition. This difference between _N_R_eq_ in cycle 1 and _N_R_eq_ in cycle 15 is considered a mathematical representation of the cumulative change in surface ligand activity over 15 cycles using each regeneration condition.

**Figure 4:**
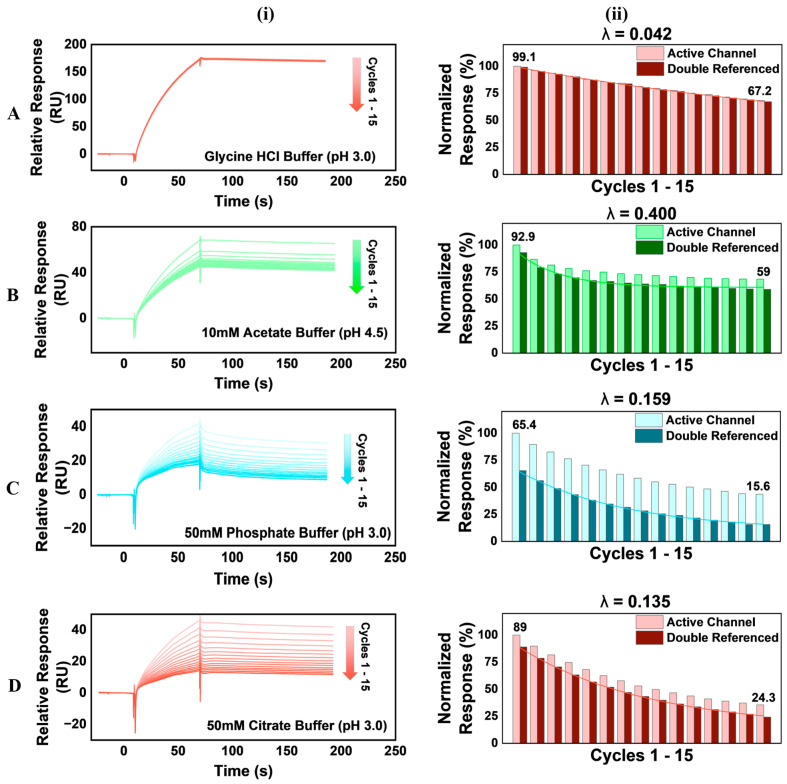
(**i**) Adjusted Regeneration verification sensorgrams and (**ii**) normalized response trends of (**A**) glycine HCl (pH 3); (**B**) 10 mM acetate buffer (pH 4.5); (**C**) 50 mM phosphate buffer (pH 3); and (**D**) 50 mM citrate buffer (pH 3). The sensorgrams have been adjusted, making the baseline report point to the origins of both the x and y axes. This allows for the comparison of sensorgram shapes across the 15 cycles in each run, representing changes to the sensor surface during the run. The normalized response trends (**ii**) show the NReq of successive AVT runs in the active flow channel and the NReq for the double-referenced responses for each cycle. A one-phase exponential decay fitting curve was calculated for the double-referenced _N_R_eq_ values for each condition, and the decay constant (λ) of the curve was indicated (Origin 2024b. OriginLab Corporation, Northampton, MA, USA).

**Figure 5:**
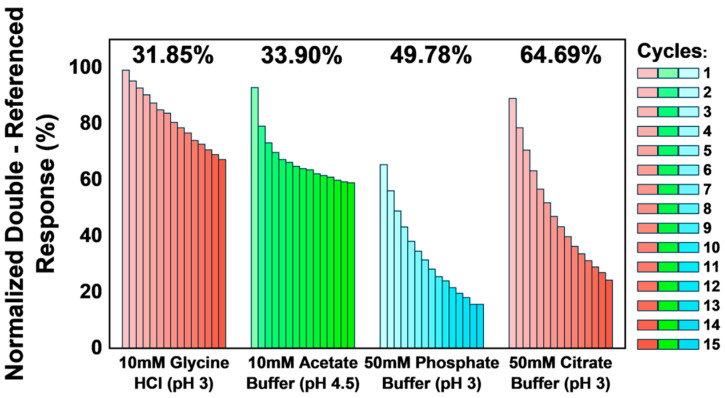
Comparisons of normalized double-referenced responses across the cycles in each regeneration condition. The difference between the double-referenced N_Req_ observed in the first and last cycles for each regeneration condition is indicated.

Sensorgrams of the sample cycles under each regeneration condition, as well as trends observed in normalized double-referenced relative responses at equilibrium, are presented in Figure 4, while a comparison of the differences observed in normalized relative responses at equilibrium across the cycles for each regeneration condition is presented in Figure 5.

The sensorgrams were obtained when glycine HCl (pH 3) was used as a regeneration condition (Figure 4A(i)), when it appears superimposed, and when minimal changes are observed in the sensorgram shape across all 15 cycles, indicating minimal modifications to the sensor surface. The regeneration condition maintains surface activity through repeated cycles with efficient surface regeneration without significant disruptions to ligand activity. This is also corroborated in the Aii plot (Figure 4), where the normalized activity declined steadily with a low decay constant (0.042), producing a 31.85% change in surface activity from cycle 1 to cycle 15 (Figure 5). With 10 mM acetate buffer (pH 4.5), we observed a significant loss of surface activity (λ = 0.4), reflecting a notable divergence in the sensorgrams obtained (Figure 4). As illustrated in Figure 5, the 10 mM acetate buffer (pH 4.5) also caused a 33.9% loss of sensor surface activity. There was a significant loss of sensor surface activity when 50 mM phosphate and 50 mM citrate buffers were used as regeneration buffers (Figure 4), with both conditions leading to 49.78% and 64.69% losses of sensor surface activities, respectively (Figure 5). Therefore, our findings indicate that 10 mM glycine buffer (pH 3) is suitable for regenerating the biotinylated FcγRI sensor surface after AVT binding. This is significant, given the previously reported difficulties regenerating the FcγRI ligand from a complex with IgG, due to the high binding affinity of both biomolecules [45,46]. Consequently, 10 mM glycine (pH 3) was used to regenerate the sensor surface between assay runs in subsequent studies.

Furthermore, we assessed sensor surface stability over 25 successive cycles, recording minimal changes to baseline response (*CV%* = 0.13%). We also fitted the VEGF response at equilibrium (Req) obtained with an exponential decay function, interpreting the low decay constant obtained (λ = 0.06) as a reliable indicator of sensor stability over at least 25 cycles with at least 10 analyte injections (Figure S1). This demonstrates the reusability and reliability of the sensor surface mediated by the stability afforded by the streptavidin–biotin capture chemistry employed for FcγRI immobilization, as well as the efficacy of the regeneration condition employed (10 mM glycine—pH 3).

### 3.3. IgG1-Type Monoclonal Antibody Capture Studies

**Figure 6:**
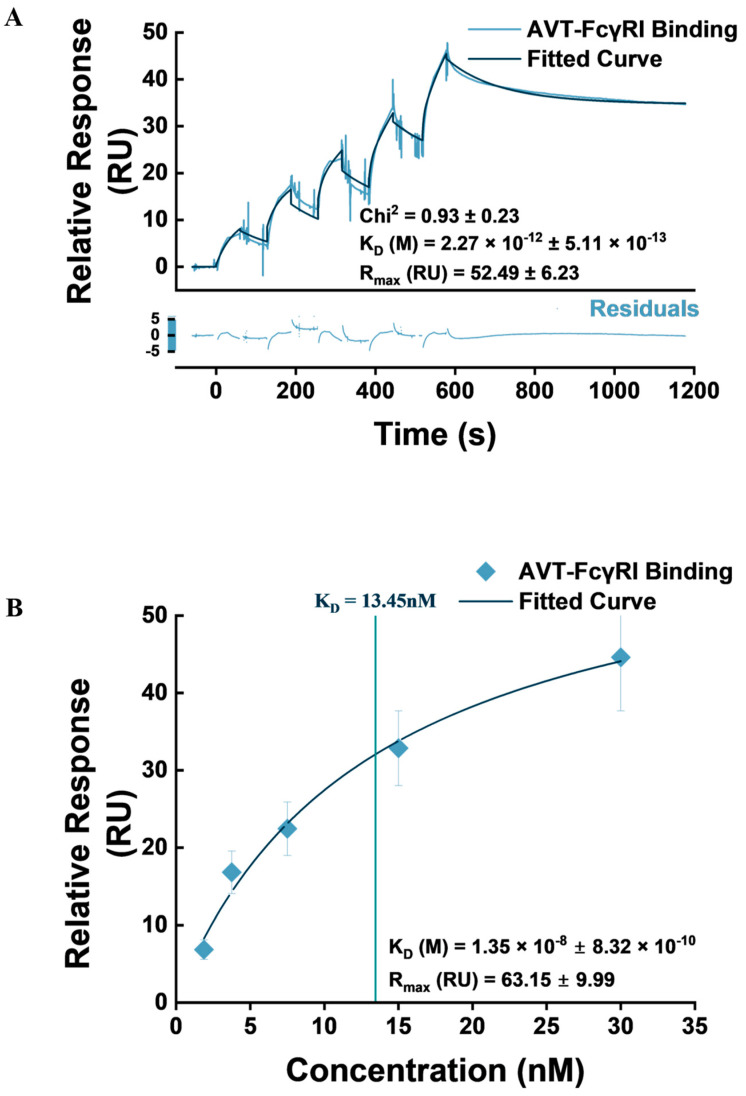
FcγRI−AVT Binding. (**A**) Representative sensorgram of AVT binding to FcγRI, fitted with the heterogeneous ligand kinetic model. (**B**) The binding affinity of AVT to FcγRI obtained by the Biacore Evaluation steady-state affinity algorithm. Sensorgrams were obtained as plots of average response from three assay repeats per data point. Steady-state affinity and kinetic constants are presented as mean ± SD.

FcγRI exhibits a high affinity for IgG1 antibodies; binding FcγRI to Fc receptors on effector cells plays a crucial role in mediating immune effector functions, effectively bridging humoral and cellular immune response [31]. Therefore, our approach to utilize a FcγRI ligand to capture the IgG1-type mAb (AVT) in the biosensor setup is based on their physiologic interactions. As has been reliably demonstrated in the literature, an SPR-based platform using label-free optical detection is ideal for studying biomolecular interactions and quantifying both steady-state binding affinity and surface-binding kinetics [47]. To achieve this, we assessed the binding response of AVT to the biotinylated FcγRI ectodomain immobilized on a streptavidin-coated SA chip. Double-referenced sensorgrams of AVT binding to FcγRI were obtained by subtracting responses from blank flow channels and zero reference cycles using the Biacore Evaluation software (Figure 6). Reference subtraction, in principle, reduces the effect of bulk shifts, baseline drifts, flow channel differences, and non-specific binding on the assay results [48].

The real-time detection of biomolecular interactions afforded by SPR bioassays enables a longitudinal collation of binding data (RU) and the onward construction of a time series curve (sensorgram), with distinct association and dissociation phases, which can then be approximated, using mathematical models, into kinetic constants that characterize the rate of attachment and detachment of an analyte biomolecule to and from the surface-immobilized ligand. These kinetic constants include the association rate constant (k_on_/k_a_), defining the rate of binding of one biomolecule to the other; the dissociation constant (k_d_/k_off_), which defines the rate of detachment of biomolecules from the complex; and an equilibrium dissociation constant (K_D_), which mathematically combines k_a_ and k_d_ to define the strength of the attachment [49]. Mathematical models adopted to derive the kinetic constants from the experimental data include the 1:1 Langmuir binding model, which defines binding between biomolecules with a simple 1:1 binding stoichiometry, and the heterogeneous ligand model, which theoretically models the binding of an analyte to an immobilized ligand with heterogeneity in its binding sites, either due to there being different molecules immobilized or due to different binding sites on the same molecule [49]. Other models include the bivalent analyte, heterogenous analyte, and two-state reaction, each with its peculiar suitability. While it is encouraged to adopt the simplest model (1:1 Langmuir), especially in biomolecular interactions with 1:1 stoichiometry, the complexities of biomolecular interactions are often not fully captured by the simple mathematical representations inherent in the models. This is an especially pertinent issue with FcγR—IgG interactions, as discussed by Forest-Nault et al., who noted that these interactions, despite being theoretically 1:1 stoichiometrically, often cannot be accurately modeled with the 1:1 Langmuir binding model [33]. Our experience analyzing FcγRI–AVT binding kinetics assay data corroborates this. The fitting of the 1:1 binding model to our data was suboptimal, reflected in the Chi^2^ value obtained and systemic deviations, observed on visual inspection of the fit. However, the best fit was with the heterogenous ligand model. Accordingly, we report the kinetic constants obtained using the heterogeneous ligand model as the most accurate representation of the observed FcγRI–AVT binding kinetics. However, for exploratory purposes, we assessed the fit of our data to other models, including the bivalent analyte and two-state reaction models. Our findings for each model are presented in Table 1. Furthermore, due to the simplicity of the 1:1 binding model and its ubiquity in the literature, we have included AVT–FcγRI binding kinetics constants obtained in our study from the 1:1 binding model in Table S1. This is to allow for an easy comparison of our findings with similar studies. It is noteworthy that while the Langmuir binding model did not fit as well as the heterogenous ligand model adopted, it still demonstrated an acceptable fit, with the chi^2^ values obtained being less than 0.1 × the recorded R_max_ values on the three repeats (Table S1) [49].

In assessing steady-state affinity, we deployed the default steady-state affinity algorithm, which provides an equilibrium dissociation constant (K_D_) mathematically expressing the affinity of the binding interaction. Using the Biacore Evaluation software, steady-state binding affinity is obtained from a curve of steady-state response (R_eq_) against concentration (C). An extrapolation from half the highest R_eq_ (where the curve flattens on the y-axis) to the concentration is recorded as K_D_. Therefore, concentrations used for steady-state binding affinity studies must be carefully selected to ensure the R_eq_ against concentration curve plateaus [49]. Based on the prior experience of our group with the FcγRI–AVT interaction [32], we selected a concentration range of 1.875–30 nM to achieve this. An illustration of the binding kinetics and steady-state affinity obtained is presented in Figure 6.

The steady-state affinity constant (K_D_) obtained in our study was in the nanomolar range, similar to what has been reported in earlier studies [32]. We hypothesize that the lower K_D_ value obtained on this occasion (13.5 ± 0.832 nM), compared to 95.02 ± 8.14 nM earlier reported by our group for his-tagged FcγRI and 100 nM as has been established in the literature, could be as a result of the relative physicochemical instability of the anti-his capture, as was suggested by Gunnarsson et al. and in an earlier study from our group [44,50]. In contrast, biotin capture provides an oriented, stable, and accessible presentation of the FcγRI ectodomain for IgG binding.

To obtain kinetic affinity (K_D_) from the heterogeneous ligand model, we sorted the two different sets of kinetic constants (k_a_^1^, k_d_^1^, k_D_^1^ / k_a_^2^, k_d_^2^, k_D_^2^) calculated by the software based on reported R_max_, reporting an average of constants from the set with average R_max_ closest to the calculated theoretical R_max_ (52.49 ± 6.23), effectively disregarding the second set of kinetic constants with R_max_ 10.01 ± 1.11 as products of non-specific binding (Figure 6). A comparison of both sets is provided in Table 2.

The heterogeneous ligand kinetic model reported FcγRI—AVT binding affinity in the picomolar range (2.27 pM), in contrast to the kinetic affinity reported earlier in our group (2.75–2.29 nM) and the literature (52 nM) [32,45]. Similarly, the simple 1:1 binding model reported affinity constants (0.42 nM) lower than those found in the referenced studies (Table S1). Our study demonstrates an affinity value higher than has ever been recorded in the literature for FcγRI–IgG1 interactions using SPR assays without prior incubation of the IgG with antigens. This is notable because even though the high (nano–picomolar range) affinity of FcγRI for IgG1-type antibodies is well established and has been demonstrated in the literature [51], such high affinity has only been achieved in an SPR setup with prior complexation of the IgG with antigens [52]. This has important implications for the biosensing application of our setup. A stable, high-affinity capture of the mAb is key for reproducibility and accuracy in antigen detection and quantification.

### 3.4. VEGF Binding Kinetics to IgG1-FcγRI Complex

**Figure 7:**
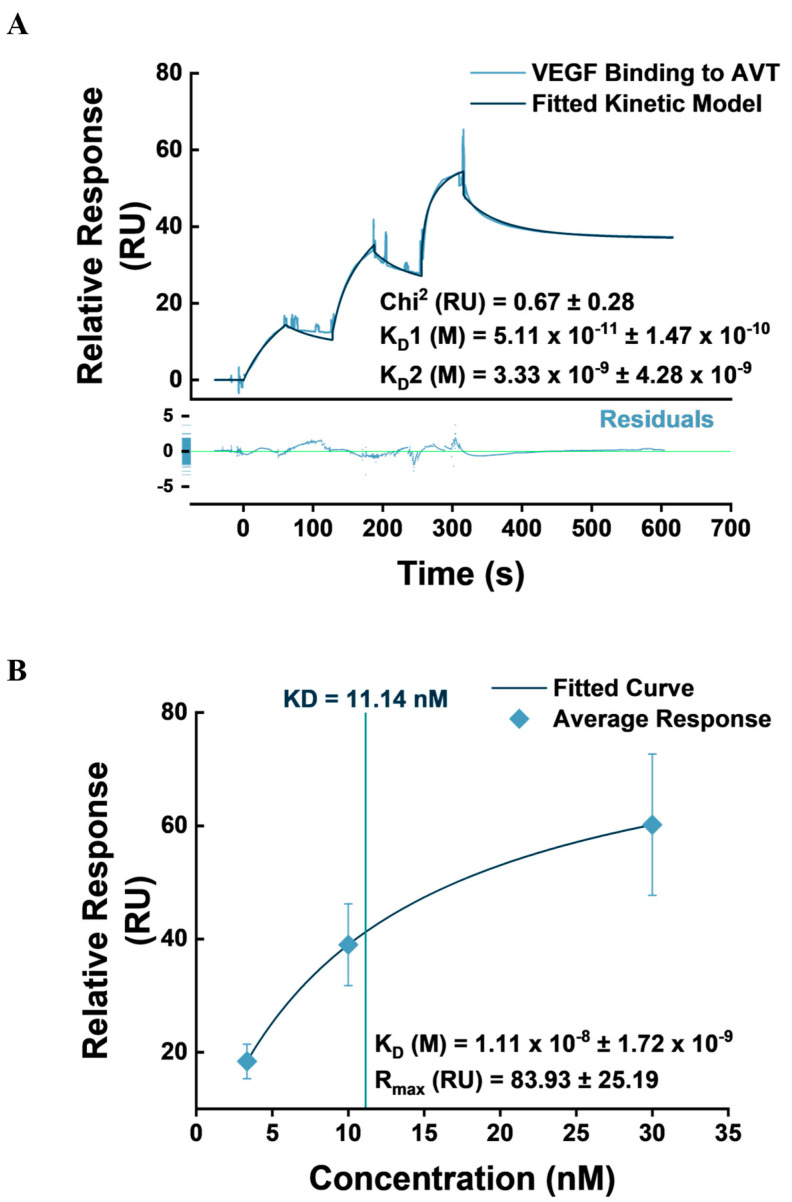
VEGF Binding to AVT. (**A**) A representative sensorgram of VEGF binding to FcγRI−captured AVT, fitted with the heterogeneous ligand kinetic model. (**B**) The binding affinity of VEGF to AVT was obtained by the Biacore Evaluation Steady-State affinity algorithm. Sensorgrams were obtained as plots of average response from nine repeats (three assay repeats on three flow channels) per data point. Steady-State affinity and kinetic constants are presented as mean ± SD.

The clinical value of AVT as an anticancer agent is predicated on its high affinity and specific interactions with VEGF, effecting the blockade of VEGF receptors 1 and 2 signaling activity, which ultimately slows down cancer cell proliferation, migration, invasiveness, cancer stem cell self-renewal, stemness maintenance, and immune suppression [39,53]. The affinity of AVT for VEGF has been reported in the literature to be in the picomolar range in various setups, including ELISA and SPR [32,54,55,56]. Accordingly, we injected VEGFA165 over the AVT-captured surface in a single-cycle kinetics assay. Data fitting was conducted with the heterogeneous ligand kinetics model, having demonstrated a better fit from visual observation and in terms of goodness of fit parameters (Chi^2^) compared to the 1:1 Langmuir binding model and the two-state reaction binding model, similar to our finding during the IgG1-type mAb capture study. Three VEGF concentrations (3.33, 10, and 30 nM) were injected in triplicate cycles over three active flow channels, providing nine datasets for the assay. A representative sensorgram of the assay repeats, fitted with the heterogeneous ligand model, and a plot of double-referenced response at equilibrium (Req) against concentration, equipped with the Biacore Evaluation steady-state algorithm, are presented in Figure 7.

The heterogenous ligand showed the best fit to the kinetic data obtained from the VEGF–AVT single-cycle kinetics assay, demonstrable from both visual observation and goodness-of-fit parameters, compared with the 1:1 and two-state reaction binding models. Additionally, the residuals plot showed randomness in distribution and remained within ±5.0 RU. Nonetheless, we report kinetic constants obtained from the 1:1 binding model in Table S2 due to its simplicity, ease of interpretation, and comparability with similar studies in the literature. Based on the output of the heterogeneous ligand model, two VEGF–AVT binding profiles are proposed, with Rmax values of 38.35 and 12.60, respectively (Table 3). The first binding profile presents a reported kinetic affinity value in the pM range, while the second binding profile reports a calculated affinity in the nanomolar range.

Notably, a high variability is observed amongst the nine datasets, reflected in standard deviations from the mean of the association, dissociation, and affinity values calculated from the heterogenous ligand model. From the 1:1 binding model, we observe this variation most notably arising from the dissociation rates (k_d_) obtained from Fc 3-1. These variations may result from differences in the sensor surface concentrations of AVT across the three active flow channels. We observed AVT capture levels ranging from 986 RU (Fc3-1) to 3020 RU (Fc4-1). Even though these capture levels have been accounted for by sensorgram normalization, baselining all sensorgram y-axes at the AVT capture level report point, there are possibilities that significant variation in kinetics findings emanate from the varying capture levels. Flow channel 4-1 with a relatively high AVT capture level, for instance, may be subject to mass transport limitations, steric hindrance, VEGF rebinding, and avidity effects. The Biacore Evaluation software (3.0 Biacore T200, Shrewsbury, MA, USA) reports a coefficient (t_c_) quantifying the mass transport component independent of the injection flow rate. We observed that the t_c_ value was consistently more than 100-fold that of the calculated association rate in each assay repeat, as recommended, potentially ruling out mass transport limitation [49]. Therefore, we hypothesize that steric, avidity effects and other complicated binding behaviors resulting from the varying AVT capture levels across flow channels may account for the wide variation in kinetic constants [57]. To address these phenomena that complicate binding kinetics, we contend that further studies might benefit from confining kinetics assays to a single flow channel, while using local fitting for the R_max_ parameter to correct for the varying capture levels between cycles and conducting multiple cycle repeats for data accuracy [49]. The biosensor setup reproduced steady-state binding affinity values similar to the literature findings [32]. The kinetic parameters calculated for the three flow channels from both binding profiles are presented in Table 3.

### 3.5. VEGF Detection and Quantification

**Figure 8:**
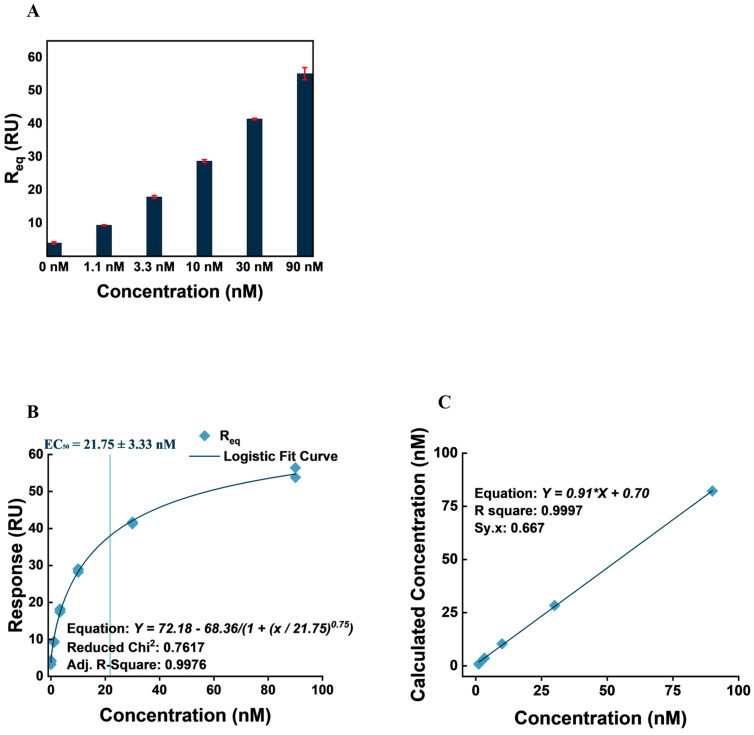
VEGF concentration analysis. (**A**) Bar plot of VEGF binding response (R_eq_) to AVT captured on a biotinylated FcγRI-immobilized surface at 0, 1.1, 3.3, 10, 30, and 90 nM. (**B**) Relative response at equilibrium (R_eq_) against concentration curve, fitted with the four-parameter logistic fitting model. The model visually fits the data plot well, as demonstrated in the low adjusted chi-squared and R-squared values. (**C**) A linearity plot demonstrating the linear correlation of concentration with the fitted model-calculated concentration.

A concentration analysis was conducted for VEGF using the biosensor setup. Five concentrations of VEGF were injected over an AVT-captured surface, and relative responses at equilibrium (Req) obtained over three repeats of each concentration over three different flow channels were computed to obtain a calibration curve. The average R_eq_ from each cycle was plotted against concentration for the three cycles, and the plot fitted with the four-parameter logistic model (Figure 8). The four-parameter logistic model has been described as a suitable model of protein–ligand interactions and biological processes. The model fits the data based on four parameters, including an upper and lower y-asymptote, EC_50_ (an extrapolated x-value for the y-value half of the upper asymptote), and a Hill’s coefficient (the slope of the curve at EC_50_) [58]. The calibration curve obtained is presented in Figure 8. As shown in Figure 8A, the calibration is well fitted to the obtained data, demonstrating a low reduced chi^2^ value (0.7617) and adjusted R-squared (0.9976). The model’s upper and lower asymptotes were 72.18 and 3.82 RU, respectively. EC_50_ was calculated as 21.75 ± 3.33 nM with a Hill’s coefficient of 0.75.

We characterized detection and quantification limits based on earlier reported mathematical methods [59]. Accordingly, we computed the critical value, also known as the limit of blank (LOB), of the biosensor setup by the VEGF concentration corresponding to ‘y_0_ + 1.645*SD’ (y_0_ being the average R_eq_ obtained from zero nM VEGF injection, and *SD* being the standard deviation [*n* = 6]) on the calibration curve; while the limit of detection (LOD) and quantification (LOQ) were computed by ‘y_0_ + 3.3*SD’ and ‘y_0_ + 10*SD’, respectively [59]. The detection and quantification values obtained with a 95% confidence level by the biosensor setup, therefore, include the following:

- LOB = 59.1 pM (2.26 µg⋅mL^−1^);
- LOD = 129.9 pM (4.96 µg⋅mL^−1^);
- LOQ = 534.6 pM (20.42 µg⋅mL^−1^).

Relatively, the setup demonstrates better detection than a similar biosensor setup developed within our research group for the detection of TNF-α using adalimumab captured with his-tagged FcγRI and protein A-coated sensor surfaces [36]. Similarly, the setup outperforms some fluorescence-based and SPR-based VEGF biosensors in the literature [60]. However, there have been lower LOD and LOQ values reported in the literature with ELISA, SPRi, and electrochemical aptasensor setups [61,62]. It is worth noting that the difference in detection limits between the current biosensor setup and others likely stems from fundamental distinctions in the principles and techniques underlying each method. ELISA, for example, is a well-established and highly sensitive method that benefits from signal amplification strategies, such as enzymatic reactions producing measurable colorimetric or fluorescent signals. These amplification steps significantly enhance sensitivity, compared to surface plasmon resonance (SPR)-based biosensors, which rely directly on the real-time measurement of mass changes at the sensor surface. Our SPR method utilizes the FcγRI-mediated immobilization of antibodies for VEGF detection. While this approach provides specificity and stability, it lacks intrinsic signal amplification mechanisms. The sensor detects mass-induced changes in the refractive index, limiting sensitivity to the direct interaction and the resulting biophysical signal. To improve the detection limit of this biosensor, several strategies could be considered. One approach would be signal amplification by the derivatization of a secondary recognition antibody with nanostructures such as gold nanoparticles and magnetic nanoparticles [63]. Another avenue could involve incorporating highly refractive multilayer thin films in the sensor chip design to enhance the electromagnetic fields of the surface plasmons [63].

Subsequently, we attempted to validate the accuracy and precision of the concentrations obtained by the calibration curve by computing calculated concentrations from responses received by sample concentrations and plotting calculated concentrations against concentrations fitted with a linear regression model. Again, the linear model demonstrated a good fit with the plot, observable visually, and from the goodness of fit values obtained (Figure 8). Additionally, accuracy/precision, as well as *CV%* values, were obtained and are presented in Table 4.

The accuracy/recovery values, as well as *CV%* values obtained (Table 4), were evaluated based on the literature recommendations of 100 ± 20% accuracy/recovery values and ≤20% *CV%* values [36,64]. The setup demonstrated acceptable accuracy/recovery, except in the lowest analyte concentration (1.11 nM), where 78.87% was recorded. As a measure of precision, the *CV%* value suggests the repeatability and reproducibility of VEGF quantification findings using the setup. *CV%* values remain lower than 6.7% throughout triplicate repeats for five concentrations over three flow channels.

### 3.6. AVT Specificity for VEGF

**Figure 9:**
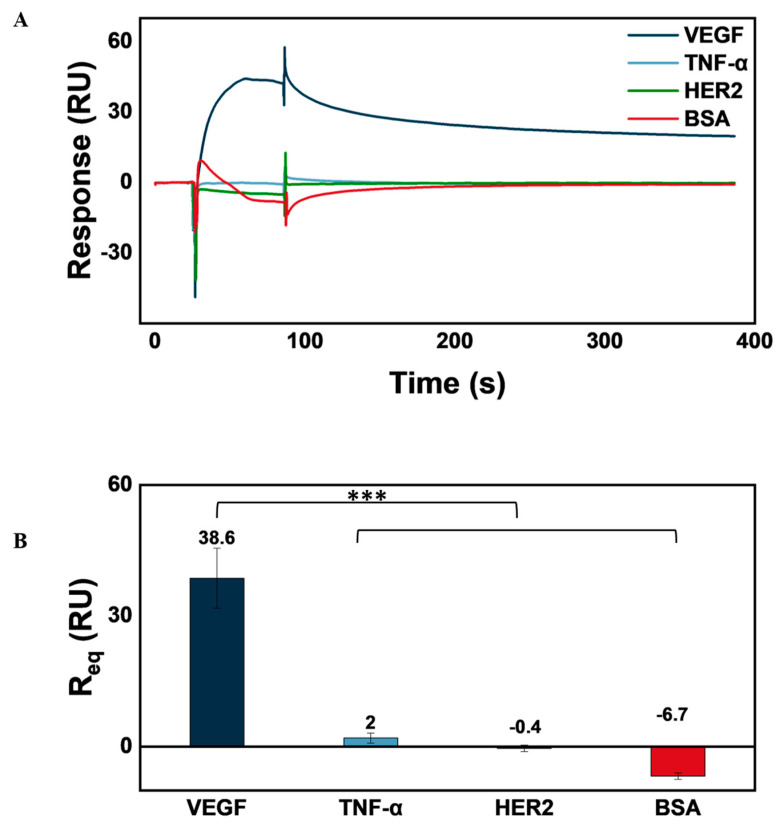
Biosensor VEGF specificity. (**A**) Sensorgram of response obtained with injections of VEGF, TNF-α, and HER2 at 30 nM, as well as BSA (0.1 mg·mL^−1^). (**B**) Average double−referenced R_eq_ obtained with each analyte (n = 9). The specificity of the biosensor setup for VEGF is demonstrated in the statistically significant average double-referenced R_eq_ obtained with VEGF, compared to other analytes (***—*p* < 0.0001).

The biosensor setup is designed to leverage the specificity of bevacizumab (AVT) for VEGF as a target antigen. To assess the recapitulation of this specificity within the biosensor, we injected different analytes, including TNF-α, HER-2, and VEGF, at 30 nM concentrations, and BSA at 0.1 mg·mL^−1^. The sensorgrams produced via the injection of each analyte as well as a plot of the double-referenced R_eq_ are presented in Figure 9.

To ascertain the statistical significance of the R_eq_ obtained from the VEGF injection of the biosensor, we compared the arrays of double-referenced R_eq_ values of VEGF against each of the other analytes, using one-tailed paired student’s *t*-tests. Obtaining p-values lower than 0.0001 validated the statistical significance of the relatively higher R_eq_ values reported with VEGF compared with the other analytes.

## 4. Conclusions

We present an optical detection setup for the label-free detection of VEGF using the SPR technique. To recapitulate biological protein interactions, we achieved the site-oriented capture of AVT on a sensor surface through its Fc interaction with immobilized FcγRI. The novelty of our approach stems from the streptavidin–biotin capture method utilized to immobilize FcγRI, providing a stable sensor surface for site-oriented AVT capture.

The high affinity of FcγRI for AVT was leveraged to capture the mAb on the sensor surface, providing kinetic affinity constants in the picomolar range, which has never been recorded in published SPR studies of FcγRI–AVT binding without prior mAb–VEGF complexation. Importantly, the controlled orientation of AVT ensures that its antigen-binding region (Fab) remains available to interact with the target analyte, VEGF. One of the key issues reported with the FcγRI biotin capture approach is sensor surface regeneration, with previous studies reporting that the high-affinity FcγRI–IgG1 binding makes regeneration either unsuccessful or destructive to ligand activity. To overcome this, regeneration scouting and verification analyses were conducted to identify the optimal reagent and condition for sensor surface regeneration, identifying 10 mM glycine hydrochloride (pH 3.0) as an appropriate solution for the effective and non-destructive regeneration of the sensor surface.

We assessed the VEGF analyte detection and quantification capacity of the AVT–based biosensor through surface-binding kinetic studies, steady-state binding affinity, and concentration analysis. A calibration curve was established, and limits of blank, detection, and quantification were calculated, with a confidence interval of 95%, as 59.1, 129.9, and 534.6 pM, respectively. The specificity of the biosensor for VEGF was also assessed by injections of sample solutions of VEGF, TNF-α, and HER2 at 30 nM, as well as BSA at 0.1 mg⋅mL^−1^. The analysis demonstrated a statistically significant (*p* < 0.01) specific response to VEGF.

The SPR-based setup introduces a novel, biomimetic approach to VEGF detection and quantification with notable differences from conventional VEGF biosensing methods. The biosensor demonstrates strong performance by leveraging FcγRI-mediated site-specific antibody capture and maintaining high affinity for VEGF detection. Its detection limits, while better than some earlier platforms within the authors’ group, remain slightly inferior to others, possibly stemming from the inherent signal constraints of traditional SPR, which, despite its high resolution, lacks the biochemical amplification present in enzymatic assays and other advanced SPR configurations. The biotin–streptavidin capture chemistry used for immobilizing FcγRI is one of the strongest non-covalent bonds, ensuring robust ligand attachment and resistance to degradation over multiple regeneration cycles, as demonstrated in our sensor stability studies. Reusability provides another strength of the platform. By employing an optimized regeneration protocol compatible with the stable biotin–streptavidin bond, we ensure that the sensor surface can be reused for multiple cycles without significant performance loss. This contrasts with conventional methods like ELISA, which is typically single-use, and offers an advantage over some other biosensing methods that lack robust regeneration capabilities. The ability to repeatedly use the same sensor surface reduces costs and enhances the practicality of this method for routine VEGF monitoring.

Generally, our findings indicate that the highly sensitive SPR setup is stable over extended periods and can detect and accurately quantify VEGF even at trace levels, providing a reliable and precise tool for biomedical diagnostics, leveraging the advantages of SPR, including label-free, real-time interaction monitoring, low reagent consumption, cost-effectiveness, and device portability. However, the setup was susceptible to limitations encountered with SPR assays, including avidity effects, analyte rebinding, and steric hindrance that complicate biomolecular interactions and make it difficult to fit them into mathematical models for kinetic rate constants calculation. This necessitates the optimization of the mAb capture level to minimize these effects. Further characterization could also be pursued, particularly in antigen selectivity within complex biological matrices, such as serum and plasma, which contain diverse biomolecules that can adsorb onto the sensor surface, leading to non-specific binding. Sensor surface chemistry could also be modified to incorporate robust antifouling coatings that minimize non-specific interactions in complex biological samples. Additionally, sensor chip design can be optimized with signal amplification functionalizations to improve the sensitivity of the setup. Overcoming these challenges could revolutionize the field, establishing this biomarker detection method as a reliable and indispensable tool in routine clinical diagnostics.

## Figures and Tables

**Table 1:** Comparison of binding kinetics model for fit to the FcγRI—AVT binding experimental data.

Binding Models	Chi^2^	Visual Inspection Comments
1:1 Langmuir Binding	2.82 ± 0.64	Poor fit with significant systemic deviations in the dissociation phase. Residuals’ distribution spans −30 to +10 RU.
Heterogenous Ligand	0.93 ± 0.23	Relatively good fit with significant overlap across association and dissociation phases. Residuals’ distribution is non-systemic and spans −5 to +5 RU.
Bivalent Analyte	3.34 ± 0.91	Poor fit with significant deviations, especially in the dissociation phase. Residuals’ distribution is non-systemic but spans −30 to +10 RU.
Two-State Reaction	2.78 ± 0.34	It’s a relatively poor fit. There are significant deviations in the dissociation phase at lower concentrations. Residuals’ distribution is systemic, spanning −30 to +10 RU

**Table 2:** Comparison of sets of kinetic constants from the heterogeneous ligand model, clustered by R_max_ values.

S/N	Chi^2^ RU	Set 1: R_max_ = 60.21 − 44.96				Set 2: R_max_ = 11.12 − 8.504			
		k_a_ M^−1^s^−1^ E+5	k_d_ s^−1^ E-7	K_D_ pM	R_max_ RU	k_a_ M^−1^s^−1^ E+5	k_d_ s^−1^ E-3	K_D_ nM	R_max_ RU
1	1.25	3.33	8.90	2.67	60.21	97	5.9	0.60	11.12
2	0.81	2.76	4.27	1.55	52.29	96	6.8	0.71	10.42
3	0.74	3.35	8.67	2.59	44.96	159	11	0.72	8.504
Mean	0.93	3.15	7.28	2.27	52.49	117	8.03	0.68	10.01
*SD*	*0.23*	*0.27*	*2.13*	*0.51*	*6.23*	*29.3*	*2.4*	*0.05*	*1.11*

**Table 3:** VEGF—AVT Binding kinetic rate constants calculated by the heterogeneous ligand model.

Flow Channel	AVT Capture Level (RU)	Binding Profile 1				Binding Profile 2			
		k_a_ M^−1^s^−1^ E+5	k_d_ s^−1^ E-7	K_D_ pM	R_max_ RU	k_a_ M^−1^s^−1^ E+5	k_d_ s^−1^ E-3	K_D_ nM	R_max_ RU
Fc 2-1	1888.17 ± 2.15	11.3 ± 0.45	20 ± 9.36	1.79 ± 0.87	30.67 ± 2.31	73.8 ± 5.62	15.5 ± 0.82	2.12 ± 0.27	16.23 ± 0.23
Fc 3-1	986.37 ± 3.65	13 ± 0.61	12.2 ± 4.22	0.96 ± 0.38	64.81 ± 5.25	108 ± 50.8	14.6 ± 12.5	1.12 ± 0.98	5.78 ± 0.97
Fc 4-1	3020.87 ± 5.15	12.3 ± 0.19	2140 ± 3610	151 ± 252	19.55 ± 3.57	131 ± 156	43.6 ± 24.9	6.76 ± 6.71	15.78 ± 1.9
Mean		12.2	724	51.1	38.35	104	24.6	3.33	12.6
*SD*		1.25	209	147	*20.7*	85.7	19.9	4.28	*5.23*

**Table 4:** Performance of AVT-captured biosensor in VEGF quantification.

Concentration (nM)	Mean Calculated Concentration (nM)	Standard Deviation	Accuracy/Recovery (%)	*CV* (*%*)
1.1	0.8667	0.0577	78.7879	6.6617
3.3	3.6333	0.2082	110.101	5.7294
10	10.3667	0.4163	103.6667	4.0161
30	28.4	0.5292	94.6667	1.8632
90	82.21	0.1556	91.3444	0.1892

## Data Availability

The raw data supporting the conclusions of this article will be made available by the authors on request.

## Supplementary Materials

The following supporting information can be downloaded at https://www.mdpi.com/article/10.3390/bios14120634/s1, Figure S1: A. Baseline stability, AVT capture level, and VEGF binding capacity of the biotinylated FcγRI immobilized streptavidin-coated chip over the course of 25 consecutive cycles with FcγRI immobilization level 200 RU, AVT concentration 30nM, and VEGF concentration 30 nM. A slight, progressive increase in baseline response was observed over the course of the study, resulting in a low coefficient of variation (*CV%*) between baseline values of 0.13% over the 25 cycles. AVT binding/capture capacity of the sensor surface, following successive injections of 30 nM AVT during each of the 25 cycles, is also illustrated as a plot of the responses obtained at equilibrium (Req). Finally, VEGF binding at stability/equilibrium (Req) of the sensor setup over the course of the study, at cycles 5, 7, 9, 11, 13, 15, 17, 19, 21, and 23 are presented as bar plots, with an exponential decay fitting function. A measurement of the Req trend following reference surface subtraction revealed minimal deterioration of the VEGF binding capacity (λ = 0.06). B. Sensorgrams obtained from the cycles of VEGF injection, adjusted with the baseline report point set to the origin of x- and y-axes. The stability of the sensor setup is demonstrated in the minimal divergence in sensorgram shapes observed across the successive cycles; Table S1: AVT binding to FcγRI kinetics constants obtained with the 1:1 binding model; Table S2: VEGF—AVT binding kinetic rate constants calculated by the 1:1 binding model.
