# Supplementary material for: Exploring the Interaction of Biotinylated FcGamma RI and IgG1 Monoclonal Antibodies on Streptavidin-Coated Plasmonic Sensor Chips for Label-Free VEGF Detection
Source: Biosensors (Basel). 2024 Dec 20;14(12):634. doi: 10.3390/bios14120634 (PMC11674972; doi:10.3390/bios14120634)
Supplement: Supplementary file 1 [file biosensors-14-00634-s001.zip › biosensors-3375441-supplementary.pdf]

biosensors

MDPI

Supplementary Data

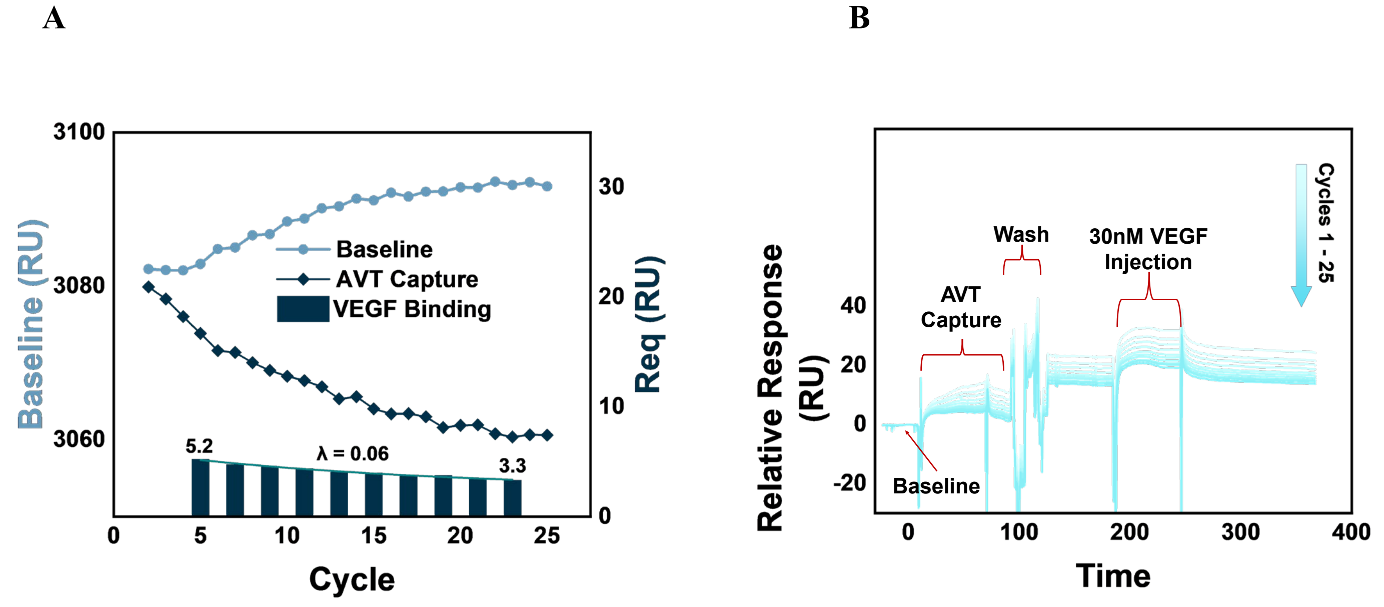

Figure S1. A. Baseline stability, AVT capture level, and VEGF binding capacity of the biotinylated FcγRI immobilized streptavidin-coated chip over the course of 25 consecutive cycles with FcγRI immobilization level 200 RU, AVT concentration 30nM, and VEGF concentration 30 nM. A slight, progressive increase in baseline response was observed over the course of the study, resulting in a low coefficient of variation (CV) between baseline values of 0.13% over the 25 cycles. AVT binding/capture capacity of the sensor surface, following successive injections of 30 nM AVT during each of the 25 cycles, is also illustrated as a plot of the responses obtained at equilibrium (Req). Finally, VEGF binding at stability/equilibrium (Req) of the sensor setup over the course of the study, at cycles 5, 7, 9, 11, 13, 15, 17, 19, 21, and 23 are presented as bar plots, with an exponential decay fitting function. A measurement of the Req trend following reference surface subtraction revealed minimal deterioration of the VEGF binding capacity (λ = 0.06). B. Sensorgrams obtained from the cycles of VEGF injection, adjusted with the baseline report point set to the origin of x and y axes. The stability of the sensor setup is demonstrated in the minimal divergence in sensorgram shapes observed across the successive cycles.

Table S1. AVT binding to FcγRI kinetics constants obtained with the 1:1 binding model.

S/N	Chi^2	k_a $M^{-1}s^{-1}$ E+5	k_d s^{-1} E-4	K_D nM	R_{max} RU
1	3.7	7.47	2.95	0.40	53.06
2	2.54	7.40	3.77	0.51	42.93
3	2.22	7.24	2.56	0.35	38.32
Mean	2.82	7.37	3.09	0.42	44.77
SD	0.64	0.097	0.51	0.066	6.16

Table S2. VEGF - AVT Binding kinetic rate constants calculated by the 1:1 binding model.

**Flow Channel**	**Binding Profile 1**			
	**k_a $M^{-1}s^{-1}$ E+5**	**k_d s^{-1} E-3**	**K_D nM**	**R_{max} RU**
Fc 2-1	17.2 ± 0.48	1.28 ± 0.13	0.74 ± 0.06	40.31 ± 1.89
Fc 3-1	14.2 ± 0.90	0.13 ± 0.09	0.09 ± 0.06	67.77 ± 6.29
Fc 4-1	15.7 ± 1.06	1.61 ± 0.13	1.03 ± 0.02	26.42 ± 1.76
Average	15.7	1.01	0.62	44.83
SD	1.5	0.68	0.42	18.54
